# Supplementary figures and images for: A descriptive study of random forest algorithm for predicting COVID-19 patients outcome
Source: PeerJ. 2020 Sep 9;8:e9945. doi: 10.7717/peerj.9945 (PMC7486830; doi:10.7717/peerj.9945)

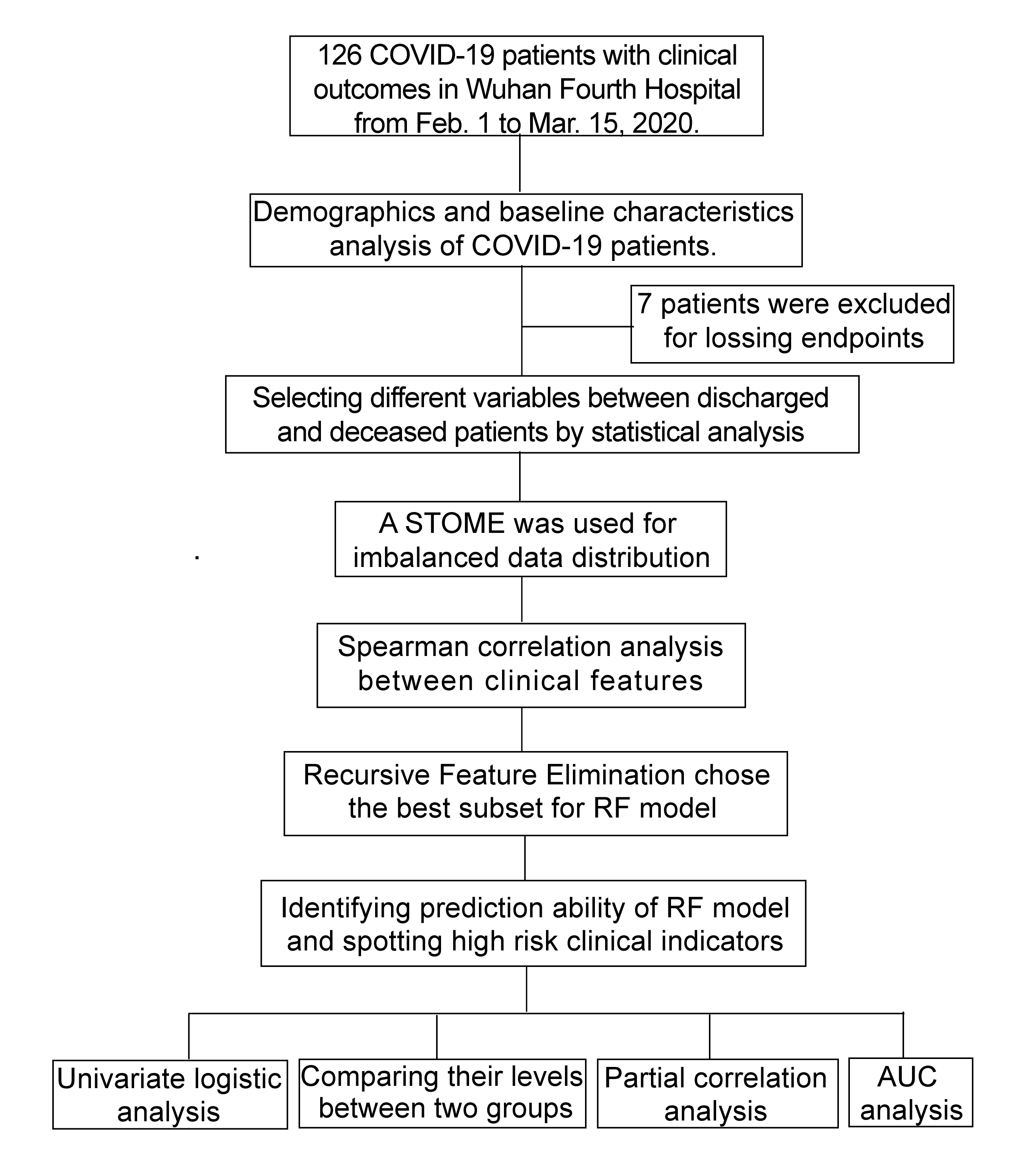

Supplement: Supplemental Information 1 [file peerj-08-9945-s001.png]
