# Supplementary material for: A descriptive study of random forest algorithm for predicting COVID-19 patients outcome
Source: PeerJ. 2020 Sep 9;8:e9945. doi: 10.7717/peerj.9945 (PMC7486830; doi:10.7717/peerj.9945)
Supplement: Supplemental Information 2 [file peerj-08-9945-s002.docx]

1.
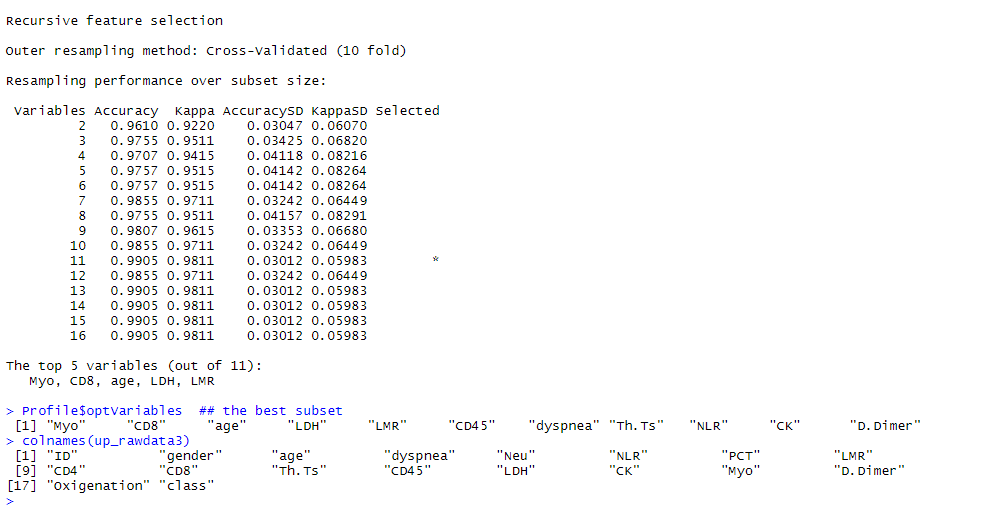
**The original data of the Recursive feature elimination (RFE) process of the feature selection with 10-fold cross-validation.**
2. **The original data of the optimal mtry selection with 5-fold cross-validation.**


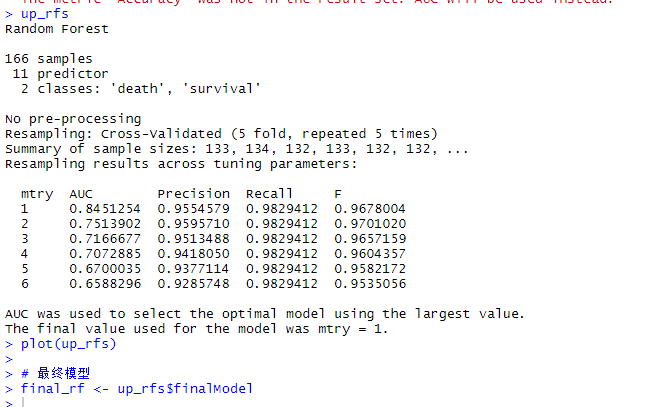


1. **The description of partial dependence correlation analysis.**

**Description**

Description Partial dependence plot gives a graphical depiction of the marginal effect of a variable on the class probability (classification) or response (regression).

**Details**

The function being plotted was defined as:

$\tilde{f}_{\left( x \right)}=\frac{1}{n}\sum_{n=1}^{n} f\left( x,x_{ic} \right)$

where x is the variable corresponding to the chosen clinical characteristic, and xiC represents the other variables in the clinical information. The summand was the predicted logits (log of a fraction of votes) for classification:

$f\left( x \right)=\log_{\mathrm{Pk}} \left( x \right)-\frac{1}{k}\sum_{j=1}^{k} \log_{Pj}\left( x \right)$

where K is the number of classes, and Pj is the proportion of votes for class j.

References:

Friedman, J. (2001). Greedy function approximation: the gradient boosting machine, Ann. of Stat.

Greenwell BM. 2017. pdp: An R package for constructing partial dependence plots. *The R Journal* 9:421-436.
