# Supplementary material for: A descriptive study of random forest algorithm for predicting COVID-19 patients outcome
Source: PeerJ. 2020 Sep 9;8:e9945. doi: 10.7717/peerj.9945 (PMC7486830; doi:10.7717/peerj.9945)
Supplement: Supplemental Information 11 [file peerj-08-9945-s011.pdf]

Package ‘corrplot’

October 17, 2017

Type Package

Title Visualization of a Correlation Matrix

Version 0.84

Date 2017-10-16

Maintainer Taiyun Wei <weitaiyun@gmail.com>

Suggests knitr, RColorBrewer, testthat

Description A graphical display of a correlation matrix or general matrix. It also contains some algorithms to do matrix reordering. In addition, corrplot is good at details, including choosing color, text labels, color labels, layout, etc.

License GPL

URL https://github.com/taiyun/corrplot

BugReports https://github.com/taiyun/corrplot/issues

VignetteBuilder knitr

RoxygenNote 6.0.1

NeedsCompilation no

Author Taiyun Wei [cre, aut],
Viliam Simko [aut],
Michael Levy [ctb],
Yihui Xie [ctb],
Yan Jin [ctb],
Jeff Zemla [ctb]

Repository CRAN

Date/Publication 2017-10-16 22:58:08 UTC

R topics documented:

corrplot-package 2
colorlegend 2
cor.mtest 4

corrMatOrder 4
corrplot 6
corrplot.mixed 13
corrRect 15
corrRect.hclust 16

Index **18**

`corrplot-package` *Visualization of a correlation matrix*

Description

The corrplot package is a graphical display of a correlation matrix, confidence interval or general matrix. It also contains some algorithms to do matrix reordering. In addition, corrplot is good at details, including choosing color, text labels, color labels, layout, etc.

Author(s)

Taiyun Wei (weitaiyun@gmail.com)

Viliam Simko (viliam.simko@gmail.com)

Maintainer: Taiyun Wei (weitaiyun@gmail.com)

References

Michael Friendly (2002). *Corrgrams: Exploratory displays for correlation matrices*. The American Statistician, 56, 316–324.

D.J. Murdoch, E.D. Chow (1996). *A graphical display of large correlation matrices*. The American Statistician, 50, 178–180.

See Also

The `plotcorr` function in the `ellipse` package and `corrgram` function in the `corrgram` package has some similarities.

`colorlegend` *Draw color legend.*

Description

Draw color legend.

Usage

```
colorlegend(colbar, labels, at = NULL, xlim = c(0, 1), ylim = c(0, 1),
  vertical = TRUE, ratio.colbar = 0.4, lim.segment = "auto",
  align = c("c", "l", "r"), addlabels = TRUE, ...)
```

Arguments

colbar	Vector, color of colbar.
labels	Vector, numeric or character to be written.
at	Numeric vector (quantile), the position to put labels. See examples for details.
xlim	See in plot
ylim	See in plot
vertical	Logical, whether the colorlegend is vertical or horizon.
ratio.colbar	The width ratio of colorbar to the total colorlegend (including colorbar, segments and labels).
lim.segment	Vector (quantile) of length 2, the elements should be in [0,1], giving segments coordinates ranges. If the value is NULL or "auto", then the ranges are derived automatically.
align	Character, alignment type of labels, "l" means left, "c" means center and "r" right.
addlabels	Logical, whether add text label or not.
...	Additional arguments, passed to plot

Author(s)

Taiyun Wei

Examples

```
par(mar = rep(0,4))
plot(0,xlim = c(0,6), ylim = c(-0.5,1.2), type = "n")

colorlegend(rainbow(100), 0:9)

colorlegend(heat.colors(100), LETTERS[1:12], xlim = c(1,2))

colorlegend(terrain.colors(100), 0:9, ratio.colbar = 0.6,
            lim.segment = c(0,0.6), xlim = c(2,3), align = "l")

colorlegend(topo.colors(100), 0:9, lim.segment = c(0,0.6),
            xlim = c(3,4), align = "l", offset = 0)

colorlegend(cm.colors(100),1:5, xlim = c(4,5))

colorlegend(sample(rainbow(12)), labels = LETTERS[1:12],
            at = seq(0.05, 0.95, len = 12), xlim = c(5,6), align = "r")

colorlegend(colbar = grey(1:100 / 100), 1:10, col = "red", align = "l",
            xlim = c(0, 6), ylim = c(-0.5,-0.1), vertical = FALSE)

colorlegend(sample(rainbow(12)),
            labels = LETTERS[1:12], at = seq(0.05, 0.95, len = 12),
            xlim = c(0, 6), ylim = c(1.1, 1.2), vertical = FALSE)
```

`cor.mtest` *Significance test which produces p-values and confidence intervals for each pair of input features.*

Description

Significance test which produces p-values and confidence intervals for each pair of input features.

Usage

```
cor.mtest(mat, ...)
```

Arguments

`mat`	Input matrix of size `FxS`, with `F` columns that represent features and `S` rows that represent samples.
`...`	Additional arguments passed to function `cor.test`, e.g. `conf.level = 0.95`.

Value

Return a list containing:

`p`	Square matrix of size `FxF` with p-values as cells
`lowCI`	Square matrix of size `FxF`, each cell represents the *lower part* of a confidence interval
`uppCI`	Square matrix of size `FxF`, each cell represents the *upper part* of a confidence interval

See Also

Function `cor.test`

`corrMatOrder` *Reorder a correlation matrix.*

Description

Draw rectangle(s) around the chart of corrrlation matrix based on the number of each cluster's members.

Usage

```
corrMatOrder(corr, order = c("AOE", "FPC", "hclust", "alphabet"),
  hclust.method = c("complete", "ward", "ward.D", "ward.D2", "single",
  "average", "mcquitty", "median", "centroid"))
```

Arguments

`corr` Correlation matrix to reorder.

`order` Character, the ordering method for the correlation matrix.

- `"AOE"` for the angular order of the eigenvectors. It is calculated from the order of the angles, a_i:

$$a_i = tan(e_{i2}/e_{i1}), if e_{i1} > 0$$

$$a_i = tan(e_{i2}/e_{i1}) + \pi, otherwise.$$

  where e_1 and e_2 are the largest two eigenvalues of matrix `corr`. See Michael Friendly (2002) for details.
- `"FPC"` for the first principal component order.
- `"hclust"` for hierarchical clustering order.
- `"alphabet"` for alphabetical order.

`hclust.method` Character, the agglomeration method to be used when `order` is `hclust`. This should be one of `"ward"`, `"ward.D"`, `"ward.D2"`, `"single"`, `"complete"`, `"average"`, `"mcquitty"`, `"median"` or `"centroid"`.

Value

Returns a single permutation vector.

Author(s)

Taiyun Wei

See Also

Package `seriation` offers more methods to reorder matrices, such as ARSA, BBURCG, BBWRCG, MDS, TSP, Chen and so forth.

Examples

```
M <- cor(mtcars)

(order.AOE <- corrMatOrder(M, order = "AOE"))
(order.FPC <- corrMatOrder(M, order = "FPC"))
(order.hc <- corrMatOrder(M, order = "hclust"))
(order.hc2 <- corrMatOrder(M, order = "hclust", hclust.method = "ward"))

M.AOE <- M[order.AOE,order.AOE]
M.FPC <- M[order.FPC,order.FPC]
M.hc  <- M[order.hc, order.hc]
M.hc2 <- M[order.hc2,order.hc2]



par(ask = TRUE)
corrplot(M)
```

```
corrplot(M.AOE)
corrplot(M.FPC)
corrplot(M.hc)

corrplot(M.hc)
corrRect.hclust(corr = M.hc, k = 2)

corrplot(M.hc)
corrRect.hclust(corr = M.hc, k = 3)

corrplot(M.hc2)
corrRect.hclust(M.hc2, k = 2, method = "ward")
```

`corrplot` *A visualization of a correlation matrix.*

Description

A graphical display of a correlation matrix, confidence interval. The details are paid great attention to. It can also visualize a general matrix by setting `is.corr = FALSE`.

Usage

```
corrplot(corr, method = c("circle", "square", "ellipse", "number", "shade",
  "color", "pie"), type = c("full", "lower", "upper"), add = FALSE,
  col = NULL, bg = "white", title = "", is.corr = TRUE, diag = TRUE,
  outline = FALSE, mar = c(0, 0, 0, 0), addgrid.col = NULL,
  addCoef.col = NULL, addCoefasPercent = FALSE, order = c("original",
  "AOE", "FPC", "hclust", "alphabet"), hclust.method = c("complete", "ward",
  "ward.D", "ward.D2", "single", "average", "mcquitty", "median", "centroid"),
  addrect = NULL, rect.col = "black", rect.lwd = 2, tl.pos = NULL,
  tl.cex = 1, tl.col = "red", tl.offset = 0.4, tl.srt = 90,
  cl.pos = NULL, cl.lim = NULL, cl.length = NULL, cl.cex = 0.8,
  cl.ratio = 0.15, cl.align.text = "c", cl.offset = 0.5, number.cex = 1,
  number.font = 2, number.digits = NULL, addshade = c("negative",
  "positive", "all"), shade.lwd = 1, shade.col = "white", p.mat = NULL,
  sig.level = 0.05, insig = c("pch", "p-value", "blank", "n", "label_sig"),
  pch = 4, pch.col = "black", pch.cex = 3, plotCI = c("n", "square",
  "circle", "rect"), lowCI.mat = NULL, uppCI.mat = NULL, na.label = "?",
  na.label.col = "black", win.asp = 1, ...)
```

Arguments

`corr`	The correlation matrix to visualize, must be square if `order` is not `"original"`. For general matrix, please using `is.corr = FALSE` to convert.
`method`	Character, the visualization method of correlation matrix to be used. Currently, it supports seven methods, named `"circle"` (default), `"square"`, `"ellipse"`, `"number"`, `"pie"`, `"shade"` and `"color"`. See examples for details.

The areas of circles or squares show the absolute value of corresponding correlation coefficients. Method `"pie"` and `"shade"` came from Michael Friendly's job (with some adjustment about the shade added on), and `"ellipse"` came from D.J. Murdoch and E.D. Chow's job, see in section References.

`type` Character, `"full"` (default), `"upper"` or `"lower"`, display full matrix, lower triangular or upper triangular matrix.

`add` Logical, if `TRUE`, the graph is added to an existing plot, otherwise a new plot is created.

`col` Vector, the color of glyphs. It is distributed uniformly in `cl.lim`. If NULL, `col` will be `colorRampPalette(col2)(200)`, see example about col2.

`bg` The background color.

`title` Character, title of the graph.

`is.corr` Logical, whether the input matrix is a correlation matrix or not. We can visualize the non-correlation matrix by setting `is.corr = FALSE`.

`diag` Logical, whether display the correlation coefficients on the principal diagonal.

`outline` Logical or character, whether plot outline of circles, square and ellipse, or the color of these glyphs. For pie, this represents the color of the circle outlining the pie. If `outline` is `TRUE`, the default value is `"black"`.

`mar` See `par`.

`addgrid.col` The color of the grid. If `NA`, don't add grid. If `NULL` the default value is chosen. The default value depends on `method`, if `method` is `color` or `shade`, the color of the grid is `NA`, that is, not draw grid; otherwise `"grey"`.

`addCoef.col` Color of coefficients added on the graph. If `NULL` (default), add no coefficients.

`addCoefasPercent`

Logic, whether translate coefficients into percentage style for spacesaving.

`order` Character, the ordering method of the correlation matrix.

- `"original"` for original order (default).
- `"AOE"` for the angular order of the eigenvectors.
- `"FPC"` for the first principal component order.
- `"hclust"` for the hierarchical clustering order.
- `"alphabet"` for alphabetical order.

See function `corrMatOrder` for details.

`hclust.method` Character, the agglomeration method to be used when `order` is `hclust`. This should be one of `"ward"`, `"ward.D"`, `"ward.D2"`, `"single"`, `"complete"`, `"average"`, `"mcquitty"`, `"median"` or `"centroid"`.

`addrect` Integer, the number of rectangles draws on the graph according to the hierarchical cluster, only valid when `order` is `hclust`. If `NULL` (default), then add no rectangles.

`rect.col` Color for rectangle border(s), only valid when `addrect` is equal or greater than 1.

`rect.lwd` Numeric, line width for borders for rectangle border(s), only valid when `addrect` is equal or greater than 1.

`tl.pos`	Character or logical, position of text labels. If character, it must be one of `"lt"`, `"ld"`, `"td"`, `"d"` or `"n"`. `"lt"`(default if `type=="full"`) means left and top, `"ld"`(default if `type=="lower"`) means left and diagonal, `"td"`(default if `type=="upper"`) means top and diagonal(near), `"d"` means diagonal, `"n"` means don't add textlabel.
`tl.cex`	Numeric, for the size of text label (variable names).
`tl.col`	The color of text label.
`tl.offset`	Numeric, for text label, see `text`.
`tl.srt`	Numeric, for text label string rotation in degrees, see `text`.
`cl.pos`	Character or logical, position of color labels; If character, it must be one of `"r"` (default if `type=="upper"` or `"full"`), `"b"` (default if `type=="lower"`) or `"n"`, `"n"` means don't draw colorlabel.
`cl.lim`	The limits `(x1, x2)` in the colorlabel.
`cl.length`	Integer, the number of number-text in colorlabel, passed to `colorlegend`. If `NULL`, `cl.length` is `length(col) + 1` when `length(col) <=20`; `cl.length` is 11 when `length(col) > 20`
`cl.cex`	Numeric, cex of number-label in colorlabel, passed to `colorlegend`.
`cl.ratio`	Numeric, to justify the width of colorlabel, 0.1~0.2 is suggested.
`cl.align.text`	Character, `"l"`, `"c"` (default) or `"r"`, for number-label in colorlabel, `"l"` means left, `"c"` means center, and `"r"` means right.
`cl.offset`	Numeric, for number-label in colorlabel, see `text`.
`number.cex`	The `cex` parameter to send to the call to `text` when writing the correlation coefficients into the plot.
`number.font`	the `font` parameter to send to the call to `text` when writing the correlation coefficients into the plot.
`number.digits`	indicating the number of decimal digits to be added into the plot. Non-negative integer or NULL, default NULL.
`addshade`	Character for shade style, `"negative"`, `"positive"` or `"all"`, only valid when `method` is `"shade"`. If `"all"`, all correlation coefficients' glyph will be shaded; if `"positive"`, only the positive will be shaded; if `"negative"`, only the negative will be shaded. Note: the angle of shade line is different, 45 degrees for positive and 135 degrees for negative.
`shade.lwd`	Numeric, the line width of shade.
`shade.col`	The color of shade line.
`p.mat`	Matrix of p-value, if `NULL`, arguments `sig.level`, `insig`, `pch`, `pch.col`, `pch.cex` is invalid.
`sig.level`	Significant level, if the p-value in `p-mat` is bigger than `sig.level`, then the corresponding correlation coefficient is regarded as insignificant. If `insig` is `"label_sig"`, this may be an increasing vector of significance levels, in which case `pch` will be used once for the highest p-value interval and multiple times (e.g. "*", "**", "***") for each lower p-value interval.

insig
: Character, specialized insignificant correlation coefficients, "pch" (default), "p-value", "blank", "n", or "label_sig". If "blank", wipe away the corresponding glyphs; if "p-value", add p-values the corresponding glyphs; if "pch", add characters (see pch for details) on corresponding glyphs; if "n", don't take any measures; if "label_sig", mark significant correlations with pch (see sig.level).

pch
: Add character on the glyphs of insignificant correlation coefficients(only valid when insig is "pch"). See par.

pch.col
: The color of pch (only valid when insig is "pch").

pch.cex
: The cex of pch (only valid when insig is "pch").

plotCI
: Character, method of ploting confidence interval. If "n", don't plot confidence interval. If "rect", plot rectangles whose upper side means upper bound and lower side means lower bound, respectively, and meanwhile correlation coefficients are also added on the rectangles. If "circle", first plot a circle with the bigger absolute bound, and then plot the smaller. Warning: if the two bounds are the same sign, the smaller circle will be wiped away, thus forming a ring. Method "square" is similar to "circle".

lowCI.mat
: Matrix of the lower bound of confidence interval.

uppCI.mat
: Matrix of the upper bound of confidence interval.

na.label
: Label to be used for rendering NA cells. Default is "?". If "square", then the cell is rendered as a square with the na.label.col color.

na.label.col
: Color used for rendering NA cells. Default is "black".

win.asp
: Aspect ration for the whole plot. Value other than 1 is currently compatible only with methods "circle" and "square".

...
: Additional arguments passing to function text for drawing text lable.

Details

corrplot function offers flexible ways to visualize correlation matrix, lower and upper bound of confidence interval matrix.

Value

(Invisibly) returns a reordered correlation matrix.

Note

Cairo and cairoDevice packages is strongly recommended to produce high-quality PNG, JPEG, TIFF bitmap files, especially for that method circle, ellipse.

Row- and column names of the input matrix are used as labels rendered in the corrplot. Plothmath expressions will be used if the name is prefixed by one of the following characters: :, = or $. For example ":alpha + beta".

Author(s)

Taiyun Wei (weitaiyun@gmail.com)

Viliam Simko (viliam.simko@gmail.com)

Michael Levy (michael.levy@healthcatalyst.com)

References

Michael Friendly (2002). *Corrgrams: Exploratory displays for correlation matrices*. The American Statistician, 56, 316–324.

D.J. Murdoch, E.D. Chow (1996). *A graphical display of large correlation matrices*. The American Statistician, 50, 178–180.

See Also

Function `plotcorr` in the `ellipse` package and `corrgram` in the `corrgram` package have some similarities.

Package `seriation` offered more methods to reorder matrices, such as ARSA, BBURCG, BB-WRCG, MDS, TSP, Chen and so forth.

Examples

```
data(mtcars)
M <- cor(mtcars)
set.seed(0)

##  different color series
col1 <- colorRampPalette(c("#7F0000", "red", "#FF7F00", "yellow", "white",
                           "cyan", "#007FFF", "blue","#00007F"))
col2 <- colorRampPalette(c("#67001F", "#B2182B", "#D6604D", "#F4A582",
                           "#FDDBC7", "#FFFFFF", "#D1E5F0", "#92C5DE",
                           "#4393C3", "#2166AC", "#053061"))
col3 <- colorRampPalette(c("red", "white", "blue"))
col4 <- colorRampPalette(c("#7F0000", "red", "#FF7F00", "yellow", "#7FFF7F",
                           "cyan", "#007FFF", "blue", "#00007F"))
wb <- c("white", "black")

par(ask = TRUE)

## different color scale and methods to display corr-matrix
corrplot(M, method = "number", col = "black", cl.pos = "n")
corrplot(M, method = "number")
corrplot(M)
corrplot(M, order = "AOE")
corrplot(M, order = "AOE", addCoef.col = "grey")

corrplot(M, order = "AOE", col = col1(20), cl.length = 21, addCoef.col = "grey")
corrplot(M, order = "AOE", col = col1(10), addCoef.col = "grey")

corrplot(M, order = "AOE", col = col2(200))
corrplot(M, order = "AOE", col = col2(200), addCoef.col = "grey")
corrplot(M, order = "AOE", col = col2(20), cl.length = 21, addCoef.col = "grey")
corrplot(M, order = "AOE", col = col2(10), addCoef.col = "grey")

corrplot(M, order = "AOE", col = col3(100))
corrplot(M, order = "AOE", col = col3(10))
```

```
corrplot(M, method = "color", col = col1(20), cl.length = 21, order = "AOE",
         addCoef.col = "grey")
corrplot(M, method = "square", col = col2(200), order = "AOE")
corrplot(M, method = "ellipse", col = col1(200), order = "AOE")
corrplot(M, method = "shade", col = col3(20), order = "AOE")
corrplot(M, method = "pie", order = "AOE")

## col = wb
corrplot(M, col = wb, order = "AOE", outline = TRUE, cl.pos = "n")

## like Chinese wiqi, suit for either on screen or white-black print.
corrplot(M, col = wb, bg = "gold2",  order = "AOE", cl.pos = "n")


## mixed methods: It's more efficient if using function "corrplot.mixed"
## circle + ellipse
corrplot(M, order = "AOE", type = "upper", tl.pos = "d")
corrplot(M, add = TRUE, type = "lower", method = "ellipse", order = "AOE",
         diag = FALSE, tl.pos = "n", cl.pos = "n")

## circle + square
corrplot(M, order = "AOE",type = "upper", tl.pos = "d")
corrplot(M, add = TRUE, type = "lower", method = "square", order = "AOE",
         diag = FALSE, tl.pos = "n", cl.pos = "n")

## circle + colorful number
corrplot(M, order = "AOE", type = "upper", tl.pos = "d")
corrplot(M, add = TRUE, type = "lower", method = "number", order = "AOE",
         diag = FALSE, tl.pos = "n", cl.pos = "n")

## circle + black number
corrplot(M, order = "AOE", type = "upper", tl.pos = "tp")
corrplot(M, add = TRUE, type = "lower", method = "number", order = "AOE",
         col = "black", diag = FALSE, tl.pos = "n", cl.pos = "n")


## order is hclust and draw rectangles
corrplot(M, order = "hclust")
corrplot(M, order = "hclust", addrect = 2)
corrplot(M, order = "hclust", addrect = 3, rect.col = "red")
corrplot(M, order = "hclust", addrect = 4, rect.col = "blue")
corrplot(M, order = "hclust", hclust.method = "ward.D2", addrect = 4)



## visualize a  matrix in [0, 1]
corrplot(abs(M), order = "AOE", cl.lim = c(0,1))
corrplot(abs(M), order = "AOE", col = col1(20), cl.lim = c(0,1))
corrplot(abs(M), order = "AOE", col = col3(200), cl.lim = c(0,1))


## visualize a  matrix in [-100, 100]
ran <- round(matrix(runif(225, -100,100), 15))
```

```
corrplot(ran, is.corr = FALSE)
corrplot(ran, is.corr = FALSE, cl.lim = c(-100, 100))


## text-labels and plot type
corrplot(M, order = "AOE", tl.srt = 45)
corrplot(M, order = "AOE", tl.srt = 60)
corrplot(M, order = "AOE", tl.pos = "d", cl.pos = "n")
corrplot(M, order = "AOE", diag = FALSE, tl.pos = "d")
corrplot(M, order = "AOE", type = "upper")
corrplot(M, order = "AOE", type = "upper", diag = FALSE)
corrplot(M, order = "AOE", type = "lower", cl.pos = "b")
corrplot(M, order = "AOE", type = "lower", cl.pos = "b", diag = FALSE)



#### color-legend
corrplot(M, order = "AOE", cl.ratio = .2, cl.align = "l")
corrplot(M, order = "AOE", cl.ratio = .2, cl.align = "c")
corrplot(M, order = "AOE", cl.ratio = .2, cl.align = "r")
corrplot(M, order = "AOE", cl.pos = "b")
corrplot(M, order = "AOE", cl.pos = "b", tl.pos = "d")
corrplot(M, order = "AOE", cl.pos = "n")


## deal with missing Values
M2 <- M
diag(M2) = NA
corrplot(M2)
corrplot(M2, na.label = "o")
corrplot(M2, na.label = "NA")


##the input matrix is not square
corrplot(M[1:8,])
corrplot(M[,1:8])


res1 <- cor.mtest(mtcars, conf.level = 0.95)
res2 <- cor.mtest(mtcars, conf.level = 0.99)


## specialized the insignificant value according to the significant level
corrplot(M, p.mat = res1$p, sig.level = 0.2)
corrplot(M, p.mat = res1$p, sig.level = 0.05)
corrplot(M, p.mat = res1$p, sig.level = 0.01)
corrplot(M, p.mat = res1$p, insig = "blank")
corrplot(M, p.mat = res1$p, insig = "p-value")
corrplot(M, p.mat = res1$p, insig = "p-value", sig.level = -1) ## add all p-values
corrplot(M, p.mat = res1$p, order = "hclust", insig = "blank", addrect = 3)
corrplot(M, p.mat = res1$p, order = "hclust", insig = "pch", addrect = 3)
```

```
## plot confidence interval(0.95), "square" method
corrplot(M, low = res1$uppCI, upp = res1$uppCI,
         plotCI = "circle", addg = "grey20", cl.pos = "n")
corrplot(M, p.mat = res1$p, low = res1$lowCI, upp = res1$uppCI,
         plotCI = "circle", addg = "grey20", cl.pos = "n")
corrplot(M, low = res1$lowCI, upp = res1$uppCI,
         col = c("white", "black"), bg = "gold2", order = "AOE",
         plotCI = "circle", cl.pos = "n", pch.col = "red")
corrplot(M, p.mat = res1$p, low = res1$lowCI, upp = res1$uppCI,
         col = c("white", "black"), bg = "gold2", order = "AOE",
         plotCI = "circle", cl.pos = "n", pch.col = "red")

## plot confidence interval(0.95), "square" method
corrplot(M, low = res1$lowCI, upp = res1$uppCI,
         col = c("white", "black"), bg = "gold2", order = "AOE",
         plotCI = "square", addg = NULL, cl.pos = "n")
corrplot(M, p.mat = res1$p, low = res1$lowCI, upp = res1$uppCI,
         col = c("white","black"), bg = "gold2", order = "AOE", pch.col = "red",
         plotCI = "square", addg = NULL, cl.pos = "n")

## plot confidence interval(0.95, 0.95, 0.99), "rect" method
corrplot(M, low = res1$lowCI, upp = res1$uppCI, order = "hclust",
         rect.col = "navy", plotCI = "rect",cl.pos = "n")
corrplot(M, p.mat = res1$p, low = res1$lowCI, upp = res1$uppCI,
         order = "hclust", pch.col = "red", sig.level = 0.05, addrect = 3,
         rect.col = "navy", plotCI = "rect", cl.pos = "n")
corrplot(M, p.mat = res2$p, low = res2$lowCI, upp = res2$uppCI,
         order = "hclust", pch.col = "red", sig.level = 0.01, addrect = 3,
         rect.col = "navy", plotCI = "rect", cl.pos = "n")


## an animation of changing confidence interval in different significance level
## begin.animaton
par(ask = FALSE)
for (i in seq(0.1, 0, -0.005)) {
  tmp <- cor.mtest(mtcars, conf.level = 1 - i)
  corrplot(M, p.mat = tmp$p, low = tmp$lowCI, upp = tmp$uppCI, order = "hclust",
           pch.col = "red", sig.level = i, plotCI = "rect", cl.pos = "n",
           mar = c(0, 0, 1, 0),
           title = substitute(alpha == x,
                              list(x = format(i, digits = 3, nsmall = 3))))
  Sys.sleep(0.15)
}
## end.animaton
```

`corrplot.mixed` *Using mixed methods to visualize a correlation matrix.*

Description

Using mixed methods to visualize a correlation matrix.

Usage

```
corrplot.mixed(corr, lower = "number", upper = "circle", tl.pos = c("d",
  "lt", "n"), diag = c("n", "l", "u"), bg = "white", addgrid.col = "grey",
  lower.col = NULL, upper.col = NULL, plotCI = c("n", "square", "circle",
  "rect"), mar = c(0, 0, 0, 0), ...)
```

Arguments

`corr`	Matrix, the correlation matrix to visualize.
`lower`	Character, the visualization method for the lower triangular correlation matrix.
`upper`	Character, the visualization method for the upper triangular correlation matrix.
`tl.pos`	Character, `"lt"`, `"d"` or `"n"`, giving position of text labels, `"lt"` means left and top, `"d"` means diagonal. If `"n"`, add no textlabel.
`diag`	Character, for specifying the glyph on the principal diagonal. It is one of `"n"` (default, draw nothing), `"l"` (draw the glyphs of lower triangular) or `"u"` (draw the glyphs of upper triangular).
`bg`	The background color.
`addgrid.col`	See the `addgrid.col` parameter in the function `corrplot`
`lower.col`	Passed as `col` parameter to the lower matrix.
`upper.col`	Passed as `col` parameter to the upper matrix.
`plotCI`	See the `plotCI` parameter in the function `corrplot`
`mar`	See `par`.
`...`	Additional arguments for corrplot's wrappers

Author(s)

Taiyun Wei

Examples

```
M <- cor(mtcars)
ord <- corrMatOrder(M, order = "AOE")
M2 <- M[ord,ord]


corrplot.mixed(M2)
corrplot.mixed(M2, lower = "ellipse", upper = "circle")
corrplot.mixed(M2, lower = "square", upper = "circle")
corrplot.mixed(M2, lower = "shade", upper = "circle")
corrplot.mixed(M2, tl.pos = "lt")
corrplot.mixed(M2, tl.pos = "lt", diag = "u")
corrplot.mixed(M2, tl.pos = "lt", diag = "l")
corrplot.mixed(M2, tl.pos = "n")
```

corrRect — *Draw rectangle(s) on the correlation matrix graph.*

Description

Draw rectangle(s) around the chart of corrrlation matrix.

Usage

```
corrRect(clus, col = "black", lwd = 2)
```

Arguments

clus	Vector, the number of each cluster's members.
col	Color of rectangles.
lwd	Line width of rectangles.

Details

`corrRect` needs the number(parameter `clus`) of each cluster's members, while `corrRect.hclust` can get the members in each cluster based on hierarchical clustering (`hclust`).

Author(s)

Taiyun Wei

Examples

```
data(mtcars)
M <- cor(mtcars)
corrplot(M, method = "circle", order = "FPC")
corrRect(c(5,6))

(order.hc <- corrMatOrder(M, order = "hclust"))
(order.hc2 <- corrMatOrder(M, order = "hclust", hclust.method = "ward"))
M.hc  <- M[order.hc, order.hc]
M.hc2 <- M[order.hc2, order.hc2]

par(ask = TRUE)

# same as: corrplot(M, order = "hclust", addrect = 2)
corrplot(M.hc)
corrRect.hclust(corr = M.hc, k = 2)

# same as: corrplot(M, order = "hclust", addrect = 3)
corrplot(M.hc)
corrRect.hclust(corr = M.hc, k = 3)

# same as: corrplot(M, order = "hclust", hclust.method = "ward", addrect = 2)
```

```
corrplot(M.hc2)
corrRect.hclust(M.hc2, k = 2, method = "ward")

# same as: corrplot(M, order = "hclust", hclust.method = "ward", addrect = 3)
corrplot(M.hc2)
corrRect.hclust(M.hc2, k = 3, method = "ward")

# same as: corrplot(M, order = "hclust", hclust.method = "ward", addrect = 4)
corrplot(M.hc2)
corrRect.hclust(M.hc2, k = 4, method = "ward")
```

---

corrRect.hclust *Draw rectangles on the correlation matrix graph.*

Description

Draw rectangles on the correlation matrix graph based on hierarchical cluster (hclust).

Usage

```
corrRect.hclust(corr, k = 2, col = "black", lwd = 2,
  method = c("complete", "ward", "ward.D", "ward.D2", "single", "average",
  "mcquitty", "median", "centroid"))
```

Arguments

corr	Correlation matrix for function corrRect.hclust. It use 1-corr as dist in hierarchical clustering (hclust).
k	Integer, the number of rectangles drawn on the graph according to the hierarchical cluster, for function corrRect.hclust.
col	Color of rectangles.
lwd	Line width of rectangles.
method	Character, the agglomeration method to be used for hierarchical clustering (hclust). This should be (an unambiguous abbreviation of) one of "ward", "ward.D", "ward.D2", "single", "complete", "average", "mcquitty", "median" or "centroid".

Author(s)

Taiyun Wei

Examples

```
data(mtcars)
M <- cor(mtcars)
corrplot(M, method = "circle", order = "FPC")
corrRect(c(5,6))

(order.hc <- corrMatOrder(M, order = "hclust"))
(order.hc2 <- corrMatOrder(M, order = "hclust", hclust.method = "ward"))
M.hc  <- M[order.hc, order.hc]
M.hc2 <- M[order.hc2, order.hc2]

par(ask = TRUE)

# same as: corrplot(M, order = "hclust", addrect = 2)
corrplot(M.hc)
corrRect.hclust(corr = M.hc, k = 2)

# same as: corrplot(M, order = "hclust", addrect = 3)
corrplot(M.hc)
corrRect.hclust(corr = M.hc, k = 3)

# same as: corrplot(M, order = "hclust", hclust.method = "ward", addrect = 2)
corrplot(M.hc2)
corrRect.hclust(M.hc2, k = 2, method = "ward")

# same as: corrplot(M, order = "hclust", hclust.method = "ward", addrect = 3)
corrplot(M.hc2)
corrRect.hclust(M.hc2, k = 3, method = "ward")

# same as: corrplot(M, order = "hclust", hclust.method = "ward", addrect = 4)
corrplot(M.hc2)
corrRect.hclust(M.hc2, k = 4, method = "ward")
```

Index

∗Topic **confidence,**
    cor.mtest, 4
∗Topic **correlation**
    corrplot-package, 2
∗Topic **correlogram**
    corrplot-package, 2
∗Topic **dimensionality**
    corrplot-package, 2
∗Topic **feature**
    corrplot-package, 2
∗Topic **hplot**
    colorlegend, 2
    corrMatOrder, 4
    corrplot-package, 2
    corrRect, 15
    corrRect.hclust, 16
∗Topic **p-value,**
    cor.mtest, 4
∗Topic **reduction**
    corrplot-package, 2
∗Topic **selection**
    corrplot-package, 2
∗Topic **significance**
    cor.mtest, 4

colorlegend, 2, *8*
cor.mtest, 4
cor.test, *4*
corrMatOrder, 4, *7*
corrplot, 6, *14*
corrplot-package, 2
corrplot.mixed, 13
corrRect, 15
corrRect.hclust, 16

hclust, *7*, *15*, *16*

par, *7*, *9*, *14*
plot, *3*

text, *8*
