# Supplementary material for: A descriptive study of random forest algorithm for predicting COVID-19 patients outcome
Source: PeerJ. 2020 Sep 9;8:e9945. doi: 10.7717/peerj.9945 (PMC7486830; doi:10.7717/peerj.9945)
Supplement: Supplemental Information 12 [file peerj-08-9945-s012.pdf]

Package ‘randomForest’

March 25, 2018

Title Breiman and Cutler's Random Forests for Classification and Regression

Version 4.6-14

Date 2018-03-22

Depends R (>= 3.2.2), stats

Suggests RColorBrewer, MASS

Author Fortran original by Leo Breiman and Adele Cutler, R port by Andy Liaw and Matthew Wiener.

Description Classification and regression based on a forest of trees using random inputs, based on Breiman (2001) <DOI:10.1023/A:1010933404324>.

Maintainer Andy Liaw <andy_liaw@merck.com>

License GPL (>= 2)

URL https://www.stat.berkeley.edu/~breiman/RandomForests/

NeedsCompilation yes

Repository CRAN

Date/Publication 2018-03-25 15:00:24 UTC

R topics documented:

classCenter 2
combine 3
getTree 4
grow 5
importance 6
imports85 7
margin 8
MDSplot 9
na.roughfix 10
outlier 11
partialPlot 12
plot.randomForest 14
predict.randomForest 15

randomForest 17
rfcv 21
rfImpute 23
rfNews 25
treesize 25
tuneRF 26
varImpPlot 27
varUsed 28

Index **29**

`classCenter` *Prototypes of groups.*

Description

Prototypes are 'representative' cases of a group of data points, given the similarity matrix among the points. They are very similar to medoids. The function is named 'classCenter' to avoid conflict with the function `prototype` in the `methods` package.

Usage

```
classCenter(x, label, prox, nNbr = min(table(label))-1)
```

Arguments

`x`	a matrix or data frame
`label`	group labels of the rows in `x`
`prox`	the proximity (or similarity) matrix, assumed to be symmetric with 1 on the diagonal and in [0, 1] off the diagonal (the order of row/column must match that of `x`)
`nNbr`	number of nearest neighbors used to find the prototypes.

Details

This version only computes one prototype per class. For each case in `x`, the `nNbr` nearest neighors are found. Then, for each class, the case that has most neighbors of that class is identified. The prototype for that class is then the medoid of these neighbors (coordinate-wise medians for numerical variables and modes for categorical variables).

This version only computes one prototype per class. In the future more prototypes may be computed (by removing the 'neighbors' used, then iterate).

Value

A data frame containing one prototype in each row.

Author(s)

Andy Liaw

See Also

randomForest, MDSplot

Examples

```
data(iris)
iris.rf <- randomForest(iris[,-5], iris[,5], prox=TRUE)
iris.p <- classCenter(iris[,-5], iris[,5], iris.rf$prox)
plot(iris[,3], iris[,4], pch=21, xlab=names(iris)[3], ylab=names(iris)[4],
     bg=c("red", "blue", "green")[as.numeric(factor(iris$Species))],
     main="Iris Data with Prototypes")
points(iris.p[,3], iris.p[,4], pch=21, cex=2, bg=c("red", "blue", "green"))
```

---

combine — *Combine Ensembles of Trees*

Description

Combine two more more ensembles of trees into one.

Usage

```
combine(...)
```

Arguments

`...`	two or more objects of class `randomForest`, to be combined into one.

Value

An object of class `randomForest`.

Note

The `confusion`, `err.rate`, `mse` and `rsq` components (as well as the corresponding components in the `test` compnent, if exist) of the combined object will be `NULL`.

Author(s)

Andy Liaw <andy_liaw@merck.com>

See Also

randomForest, grow

Examples

```
data(iris)
rf1 <- randomForest(Species ~ ., iris, ntree=50, norm.votes=FALSE)
rf2 <- randomForest(Species ~ ., iris, ntree=50, norm.votes=FALSE)
rf3 <- randomForest(Species ~ ., iris, ntree=50, norm.votes=FALSE)
rf.all <- combine(rf1, rf2, rf3)
print(rf.all)
```

---

getTree *Extract a single tree from a forest.*

Description

This function extract the structure of a tree from a `randomForest` object.

Usage

```
getTree(rfobj, k=1, labelVar=FALSE)
```

Arguments

`rfobj`	a `randomForest` object.
`k`	which tree to extract?
`labelVar`	Should better labels be used for splitting variables and predicted class?

Details

For numerical predictors, data with values of the variable less than or equal to the splitting point go to the left daughter node.

For categorical predictors, the splitting point is represented by an integer, whose binary expansion gives the identities of the categories that goes to left or right. For example, if a predictor has four categories, and the split point is 13. The binary expansion of 13 is (1, 0, 1, 1) (because $13 = 1 * 2^0 + 0 * 2^1 + 1 * 2^2 + 1 * 2^3$), so cases with categories 1, 3, or 4 in this predictor get sent to the left, and the rest to the right.

Value

A matrix (or data frame, if `labelVar=TRUE`) with six columns and number of rows equal to total number of nodes in the tree. The six columns are:

`left daughter`	the row where the left daughter node is; 0 if the node is terminal
`right daughter`	the row where the right daughter node is; 0 if the node is terminal
`split var`	which variable was used to split the node; 0 if the node is terminal
`split point`	where the best split is; see Details for categorical predictor
`status`	is the node terminal (-1) or not (1)
`prediction`	the prediction for the node; 0 if the node is not terminal

Author(s)

Andy Liaw <andy_liaw@merck.com>

See Also

randomForest

Examples

```
data(iris)
## Look at the third trees in the forest.
getTree(randomForest(iris[,-5], iris[,5], ntree=10), 3, labelVar=TRUE)
```

---

grow *Add trees to an ensemble*

Description

Add additional trees to an existing ensemble of trees.

Usage

```
## S3 method for class 'randomForest'
grow(x, how.many, ...)
```

Arguments

x	an object of class randomForest, which contains a forest component.
how.many	number of trees to add to the randomForest object.
...	currently ignored.

Value

An object of class randomForest, containing how.many additional trees.

Note

The confusion, err.rate, mse and rsq components (as well as the corresponding components in the test compnent, if exist) of the combined object will be NULL.

Author(s)

Andy Liaw <andy_liaw@merck.com>

See Also

combine, randomForest

Examples

```
data(iris)
iris.rf <- randomForest(Species ~ ., iris, ntree=50, norm.votes=FALSE)
iris.rf <- grow(iris.rf, 50)
print(iris.rf)
```

---

importance *Extract variable importance measure*

Description

This is the extractor function for variable importance measures as produced by `randomForest`.

Usage

```
## S3 method for class 'randomForest'
importance(x, type=NULL, class=NULL, scale=TRUE, ...)
```

Arguments

`x`	an object of class `randomForest`.
`type`	either 1 or 2, specifying the type of importance measure (1=mean decrease in accuracy, 2=mean decrease in node impurity).
`class`	for classification problem, which class-specific measure to return.
`scale`	For permutation based measures, should the measures be divided their "standard errors"?
`...`	not used.

Details

Here are the definitions of the variable importance measures. The first measure is computed from permuting OOB data: For each tree, the prediction error on the out-of-bag portion of the data is recorded (error rate for classification, MSE for regression). Then the same is done after permuting each predictor variable. The difference between the two are then averaged over all trees, and normalized by the standard deviation of the differences. If the standard deviation of the differences is equal to 0 for a variable, the division is not done (but the average is almost always equal to 0 in that case).

The second measure is the total decrease in node impurities from splitting on the variable, averaged over all trees. For classification, the node impurity is measured by the Gini index. For regression, it is measured by residual sum of squares.

Value

A matrix of importance measure, one row for each predictor variable. The column(s) are different importance measures.

See Also

randomForest, varImpPlot

Examples

```
set.seed(4543)
data(mtcars)
mtcars.rf <- randomForest(mpg ~ ., data=mtcars, ntree=1000,
                          keep.forest=FALSE, importance=TRUE)
importance(mtcars.rf)
importance(mtcars.rf, type=1)
```

---

imports85 *The Automobile Data*

Description

This is the 'Automobile' data from the UCI Machine Learning Repository.

Usage

```
data(imports85)
```

Format

imports85 is a data frame with 205 cases (rows) and 26 variables (columns). This data set consists of three types of entities: (a) the specification of an auto in terms of various characteristics, (b) its assigned insurance risk rating, (c) its normalized losses in use as compared to other cars. The second rating corresponds to the degree to which the auto is more risky than its price indicates. Cars are initially assigned a risk factor symbol associated with its price. Then, if it is more risky (or less), this symbol is adjusted by moving it up (or down) the scale. Actuarians call this process 'symboling'. A value of +3 indicates that the auto is risky, -3 that it is probably pretty safe.

The third factor is the relative average loss payment per insured vehicle year. This value is normalized for all autos within a particular size classification (two-door small, station wagons, sports/speciality, etc...), and represents the average loss per car per year.

Author(s)

Andy Liaw

Source

Originally created by Jeffrey C. Schlimmer, from 1985 Model Import Car and Truck Specifications, 1985 Ward's Automotive Yearbook, Personal Auto Manuals, Insurance Services Office, and Insurance Collision Report, Insurance Institute for Highway Safety.

The original data is at http://www.ics.uci.edu/~mlearn/MLSummary.html.

References

1985 Model Import Car and Truck Specifications, 1985 Ward's Automotive Yearbook.

Personal Auto Manuals, Insurance Services Office, 160 Water Street, New York, NY 10038

Insurance Collision Report, Insurance Institute for Highway Safety, Watergate 600, Washington, DC 20037

See Also

randomForest

Examples

```
data(imports85)
imp85 <- imports85[,-2]  # Too many NAs in normalizedLosses.
imp85 <- imp85[complete.cases(imp85), ]
## Drop empty levels for factors.
imp85[] <- lapply(imp85, function(x) if (is.factor(x)) x[, drop=TRUE] else x)

stopifnot(require(randomForest))
price.rf <- randomForest(price ~ ., imp85, do.trace=10, ntree=100)
print(price.rf)
numDoors.rf <- randomForest(numOfDoors ~ ., imp85, do.trace=10, ntree=100)
print(numDoors.rf)
```

---

margin *Margins of randomForest Classifier*

Description

Compute or plot the margin of predictions from a randomForest classifier.

Usage

```
## S3 method for class 'randomForest'
margin(x, ...)
## Default S3 method:
margin(x, observed, ...)
## S3 method for class 'margin'
plot(x, sort=TRUE, ...)
```

Arguments

`x`	an object of class `randomForest`, whose `type` is not `regression`, or a matrix of predicted probabilities, one column per class and one row per observation. For the `plot` method, `x` should be an object returned by `margin`.
`observed`	the true response corresponding to the data in `x`.
`sort`	Should the data be sorted by their class labels?
`...`	other graphical parameters to be passed to `plot.default`.

Value

For `margin`, the *margin* of observations from the `randomForest` classifier (or whatever classifier that produced the predicted probability matrix given to `margin`). The margin of a data point is defined as the proportion of votes for the correct class minus maximum proportion of votes for the other classes. Thus under majority votes, positive margin means correct classification, and vice versa.

Author(s)

Robert Gentlemen, with slight modifications by Andy Liaw

See Also

`randomForest`

Examples

```
set.seed(1)
data(iris)
iris.rf <- randomForest(Species ~ ., iris, keep.forest=FALSE)
plot(margin(iris.rf))
```

`MDSplot` *Multi-dimensional Scaling Plot of Proximity matrix from randomForest*

Description

Plot the scaling coordinates of the proximity matrix from randomForest.

Usage

```
MDSplot(rf, fac, k=2, palette=NULL, pch=20, ...)
```

Arguments

`rf`	an object of class `randomForest` that contains the `proximity` component.
`fac`	a factor that was used as response to train `rf`.
`k`	number of dimensions for the scaling coordinates.
`palette`	colors to use to distinguish the classes; length must be the equal to the number of levels.
`pch`	plotting symbols to use.
`...`	other graphical parameters.

Value

The output of `cmdscale` on 1 - `rf$proximity` is returned invisibly.

Note

If `k > 2`, `pairs` is used to produce the scatterplot matrix of the coordinates.

Author(s)

Robert Gentleman, with slight modifications by Andy Liaw

See Also

`randomForest`

Examples

```
set.seed(1)
data(iris)
iris.rf <- randomForest(Species ~ ., iris, proximity=TRUE,
                        keep.forest=FALSE)
MDSplot(iris.rf, iris$Species)
## Using different symbols for the classes:
MDSplot(iris.rf, iris$Species, palette=rep(1, 3), pch=as.numeric(iris$Species))
```

---

na.roughfix *Rough Imputation of Missing Values*

Description

Impute Missing Values by median/mode.

Usage

```
na.roughfix(object, ...)
```

Arguments

`object`	a data frame or numeric matrix.
`...`	further arguments special methods could require.

Value

A completed data matrix or data frame. For numeric variables, `NA`s are replaced with column medians. For factor variables, `NA`s are replaced with the most frequent levels (breaking ties at random). If `object` contains no `NA`s, it is returned unaltered.

Note

This is used as a starting point for imputing missing values by random forest.

Author(s)

Andy Liaw

See Also

`rfImpute`, `randomForest`.

Examples

```
data(iris)
iris.na <- iris
set.seed(111)
## artificially drop some data values.
for (i in 1:4) iris.na[sample(150, sample(20)), i] <- NA
iris.roughfix <- na.roughfix(iris.na)
iris.narf <- randomForest(Species ~ ., iris.na, na.action=na.roughfix)
print(iris.narf)
```

`outlier` *Compute outlying measures*

Description

Compute outlying measures based on a proximity matrix.

Usage

```
## Default S3 method:
outlier(x, cls=NULL, ...)
## S3 method for class 'randomForest'
outlier(x, ...)
```

Arguments

`x`	a proximity matrix (a square matrix with 1 on the diagonal and values between 0 and 1 in the off-diagonal positions); or an object of class `randomForest`, whose `type` is not `regression`.
`cls`	the classes the rows in the proximity matrix belong to. If not given, all data are assumed to come from the same class.
`...`	arguments for other methods.

Value

A numeric vector containing the outlying measures. The outlying measure of a case is computed as n / sum(squared proximity), normalized by subtracting the median and divided by the MAD, within each class.

See Also

randomForest

Examples

```
set.seed(1)
iris.rf <- randomForest(iris[,-5], iris[,5], proximity=TRUE)
plot(outlier(iris.rf), type="h",
     col=c("red", "green", "blue")[as.numeric(iris$Species)])
```

---

partialPlot *Partial dependence plot*

Description

Partial dependence plot gives a graphical depiction of the marginal effect of a variable on the class probability (classification) or response (regression).

Usage

```
## S3 method for class 'randomForest'
partialPlot(x, pred.data, x.var, which.class,
      w, plot = TRUE, add = FALSE,
      n.pt = min(length(unique(pred.data[, xname])), 51),
      rug = TRUE, xlab=deparse(substitute(x.var)), ylab="",
      main=paste("Partial Dependence on", deparse(substitute(x.var))),
      ...)
```

Arguments

`x`	an object of class `randomForest`, which contains a `forest` component.
`pred.data`	a data frame used for contructing the plot, usually the training data used to contruct the random forest.
`x.var`	name of the variable for which partial dependence is to be examined.
`which.class`	For classification data, the class to focus on (default the first class).
`w`	weights to be used in averaging; if not supplied, mean is not weighted
`plot`	whether the plot should be shown on the graphic device.
`add`	whether to add to existing plot (`TRUE`).
`n.pt`	if `x.var` is continuous, the number of points on the grid for evaluating partial dependence.
`rug`	whether to draw hash marks at the bottom of the plot indicating the deciles of `x.var`.
`xlab`	label for the x-axis.
`ylab`	label for the y-axis.
`main`	main title for the plot.
`...`	other graphical parameters to be passed on to `plot` or `lines`.

Details

The function being plotted is defined as:

$$\tilde{f}(x) = \frac{1}{n} \sum_{i=1}^{n} f(x, x_{iC}),$$

where x is the variable for which partial dependence is sought, and x_{iC} is the other variables in the data. The summand is the predicted regression function for regression, and logits (i.e., log of fraction of votes) for `which.class` for classification:

$$f(x) = \log p_k(x) - \frac{1}{K} \sum_{j=1}^{K} \log p_j(x),$$

where K is the number of classes, k is `which.class`, and p_j is the proportion of votes for class j.

Value

A list with two components: `x` and `y`, which are the values used in the plot.

Note

The `randomForest` object must contain the `forest` component; i.e., created with `randomForest(..., keep.forest=TRU`

This function runs quite slow for large data sets.

Author(s)

Andy Liaw `<andy\_liaw@merck.com>`

References

Friedman, J. (2001). Greedy function approximation: the gradient boosting machine, *Ann. of Stat.*

See Also

`randomForest`

Examples

```
data(iris)
set.seed(543)
iris.rf <- randomForest(Species~., iris)
partialPlot(iris.rf, iris, Petal.Width, "versicolor")

## Looping over variables ranked by importance:
data(airquality)
airquality <- na.omit(airquality)
set.seed(131)
ozone.rf <- randomForest(Ozone ~ ., airquality, importance=TRUE)
imp <- importance(ozone.rf)
impvar <- rownames(imp)[order(imp[, 1], decreasing=TRUE)]
```

```
op <- par(mfrow=c(2, 3))
for (i in seq_along(impvar)) {
    partialPlot(ozone.rf, airquality, impvar[i], xlab=impvar[i],
                main=paste("Partial Dependence on", impvar[i]),
                ylim=c(30, 70))
}
par(op)
```

`plot.randomForest` *Plot method for randomForest objects*

Description

Plot the error rates or MSE of a randomForest object

Usage

```
## S3 method for class 'randomForest'
plot(x, type="l", main=deparse(substitute(x)), ...)
```

Arguments

`x`	an object of class `randomForest`.
`type`	type of plot.
`main`	main title of the plot.
`...`	other graphical parameters.

Value

Invisibly, the error rates or MSE of the `randomForest` object. If the object has a non-null `test` component, then the returned object is a matrix where the first column is the out-of-bag estimate of error, and the second column is for the test set.

Note

This function does not work for `randomForest` objects that have `type=unsupervised`.

If the `x` has a non-null `test` component, then the test set errors are also plotted.

Author(s)

Andy Liaw

See Also

`randomForest`

Examples

```
data(mtcars)
plot(randomForest(mpg ~ ., mtcars, keep.forest=FALSE, ntree=100), log="y")
```

---

predict.randomForest *predict method for random forest objects*

Description

Prediction of test data using random forest.

Usage

```
## S3 method for class 'randomForest'
predict(object, newdata, type="response",
  norm.votes=TRUE, predict.all=FALSE, proximity=FALSE, nodes=FALSE,
  cutoff, ...)
```

Arguments

`object`	an object of class `randomForest`, as that created by the function `randomForest`.
`newdata`	a data frame or matrix containing new data. (Note: If not given, the out-of-bag prediction in `object` is returned.
`type`	one of `response`, `prob.` or `votes`, indicating the type of output: predicted values, matrix of class probabilities, or matrix of vote counts. `class` is allowed, but automatically converted to "response", for backward compatibility.
`norm.votes`	Should the vote counts be normalized (i.e., expressed as fractions)? Ignored if `object$type` is `regression`.
`predict.all`	Should the predictions of all trees be kept?
`proximity`	Should proximity measures be computed? An error is issued if `object$type` is `regression`.
`nodes`	Should the terminal node indicators (an n by ntree matrix) be return? If so, it is in the "nodes" attribute of the returned object.
`cutoff`	(Classification only) A vector of length equal to number of classes. The 'winning' class for an observation is the one with the maximum ratio of proportion of votes to cutoff. Default is taken from the `forest$cutoff` component of `object` (i.e., the setting used when running `randomForest`).
`...`	not used currently.

Value

If `object$type` is `regression`, a vector of predicted values is returned. If `predict.all=TRUE`, then the returned object is a list of two components: `aggregate`, which is the vector of predicted values by the forest, and `individual`, which is a matrix where each column contains prediction by a tree in the forest.

If `object$type` is `classification`, the object returned depends on the argument `type`:

`response` predicted classes (the classes with majority vote).

`prob` matrix of class probabilities (one column for each class and one row for each input).

`vote` matrix of vote counts (one column for each class and one row for each new input); either in raw counts or in fractions (if `norm.votes=TRUE`).

If `predict.all=TRUE`, then the `individual` component of the returned object is a character matrix where each column contains the predicted class by a tree in the forest.

If `proximity=TRUE`, the returned object is a list with two components: `pred` is the prediction (as described above) and `proximity` is the proximitry matrix. An error is issued if `object$type` is `regression`.

If `nodes=TRUE`, the returned object has a “nodes” attribute, which is an n by ntree matrix, each column containing the node number that the cases fall in for that tree.

NOTE: If the `object` inherits from `randomForest.formula`, then any data with `NA` are silently omitted from the prediction. The returned value will contain `NA` correspondingly in the aggregated and individual tree predictions (if requested), but not in the proximity or node matrices.

NOTE2: Any ties are broken at random, so if this is undesirable, avoid it by using odd number `ntree` in `randomForest()`.

Author(s)

Andy Liaw <andy_liaw@merck.com> and Matthew Wiener <matthew_wiener@merck.com>, based on original Fortran code by Leo Breiman and Adele Cutler.

References

Breiman, L. (2001), *Random Forests*, Machine Learning 45(1), 5-32.

See Also

`randomForest`

Examples

```
data(iris)
set.seed(111)
ind <- sample(2, nrow(iris), replace = TRUE, prob=c(0.8, 0.2))
iris.rf <- randomForest(Species ~ ., data=iris[ind == 1,])
iris.pred <- predict(iris.rf, iris[ind == 2,])
table(observed = iris[ind==2, "Species"], predicted = iris.pred)
## Get prediction for all trees.
```

```
predict(iris.rf, iris[ind == 2,], predict.all=TRUE)
## Proximities.
predict(iris.rf, iris[ind == 2,], proximity=TRUE)
## Nodes matrix.
str(attr(predict(iris.rf, iris[ind == 2,], nodes=TRUE), "nodes"))
```

---

randomForest *Classification and Regression with Random Forest*

Description

randomForest implements Breiman's random forest algorithm (based on Breiman and Cutler's original Fortran code) for classification and regression. It can also be used in unsupervised mode for assessing proximities among data points.

Usage

```
## S3 method for class 'formula'
randomForest(formula, data=NULL, ..., subset, na.action=na.fail)
## Default S3 method:
randomForest(x, y=NULL,  xtest=NULL, ytest=NULL, ntree=500,
             mtry=if (!is.null(y) && !is.factor(y))
             max(floor(ncol(x)/3), 1) else floor(sqrt(ncol(x))),
             replace=TRUE, classwt=NULL, cutoff, strata,
             sampsize = if (replace) nrow(x) else ceiling(.632*nrow(x)),
             nodesize = if (!is.null(y) && !is.factor(y)) 5 else 1,
             maxnodes = NULL,
             importance=FALSE, localImp=FALSE, nPerm=1,
             proximity, oob.prox=proximity,
             norm.votes=TRUE, do.trace=FALSE,
             keep.forest=!is.null(y) && is.null(xtest), corr.bias=FALSE,
             keep.inbag=FALSE, ...)
## S3 method for class 'randomForest'
print(x, ...)
```

Arguments

data	an optional data frame containing the variables in the model. By default the variables are taken from the environment which randomForest is called from.
subset	an index vector indicating which rows should be used. (NOTE: If given, this argument must be named.)
na.action	A function to specify the action to be taken if NAs are found. (NOTE: If given, this argument must be named.)
x, formula	a data frame or a matrix of predictors, or a formula describing the model to be fitted (for the print method, an randomForest object).
y	A response vector. If a factor, classification is assumed, otherwise regression is assumed. If omitted, randomForest will run in unsupervised mode.

`xtest`	a data frame or matrix (like `x`) containing predictors for the test set.
`ytest`	response for the test set.
`ntree`	Number of trees to grow. This should not be set to too small a number, to ensure that every input row gets predicted at least a few times.
`mtry`	Number of variables randomly sampled as candidates at each split. Note that the default values are different for classification (sqrt(p) where p is number of variables in `x`) and regression (p/3)
`replace`	Should sampling of cases be done with or without replacement?
`classwt`	Priors of the classes. Need not add up to one. Ignored for regression.
`cutoff`	(Classification only) A vector of length equal to number of classes. The 'winning' class for an observation is the one with the maximum ratio of proportion of votes to cutoff. Default is 1/k where k is the number of classes (i.e., majority vote wins).
`strata`	A (factor) variable that is used for stratified sampling.
`sampsize`	Size(s) of sample to draw. For classification, if sampsize is a vector of the length the number of strata, then sampling is stratified by strata, and the elements of sampsize indicate the numbers to be drawn from the strata.
`nodesize`	Minimum size of terminal nodes. Setting this number larger causes smaller trees to be grown (and thus take less time). Note that the default values are different for classification (1) and regression (5).
`maxnodes`	Maximum number of terminal nodes trees in the forest can have. If not given, trees are grown to the maximum possible (subject to limits by `nodesize`). If set larger than maximum possible, a warning is issued.
`importance`	Should importance of predictors be assessed?
`localImp`	Should casewise importance measure be computed? (Setting this to `TRUE` will override `importance`.)
`nPerm`	Number of times the OOB data are permuted per tree for assessing variable importance. Number larger than 1 gives slightly more stable estimate, but not very effective. Currently only implemented for regression.
`proximity`	Should proximity measure among the rows be calculated?
`oob.prox`	Should proximity be calculated only on "out-of-bag" data?
`norm.votes`	If `TRUE` (default), the final result of votes are expressed as fractions. If `FALSE`, raw vote counts are returned (useful for combining results from different runs). Ignored for regression.
`do.trace`	If set to `TRUE`, give a more verbose output as `randomForest` is run. If set to some integer, then running output is printed for every `do.trace` trees.
`keep.forest`	If set to `FALSE`, the forest will not be retained in the output object. If `xtest` is given, defaults to `FALSE`.
`corr.bias`	perform bias correction for regression? Note: Experimental. Use at your own risk.
`keep.inbag`	Should an `n` by `ntree` matrix be returned that keeps track of which samples are "in-bag" in which trees (but not how many times, if sampling with replacement)
`...`	optional parameters to be passed to the low level function `randomForest.default`.

Value

An object of class randomForest, which is a list with the following components:

- `call` — the original call to randomForest
- `type` — one of regression, classification, or unsupervised.
- `predicted` — the predicted values of the input data based on out-of-bag samples.
- `importance` — a matrix with nclass + 2 (for classification) or two (for regression) columns. For classification, the first nclass columns are the class-specific measures computed as mean descrease in accuracy. The nclass + 1st column is the mean descrease in accuracy over all classes. The last column is the mean decrease in Gini index. For Regression, the first column is the mean decrease in accuracy and the second the mean decrease in MSE. If importance=FALSE, the last measure is still returned as a vector.
- `importanceSD` — The "standard errors" of the permutation-based importance measure. For classification, a p by nclass + 1 matrix corresponding to the first nclass + 1 columns of the importance matrix. For regression, a length p vector.
- `localImp` — a p by n matrix containing the casewise importance measures, the [i,j] element of which is the importance of i-th variable on the j-th case. NULL if localImp=FALSE.
- `ntree` — number of trees grown.
- `mtry` — number of predictors sampled for spliting at each node.
- `forest` — (a list that contains the entire forest; NULL if randomForest is run in unsupervised mode or if keep.forest=FALSE.
- `err.rate` — (classification only) vector error rates of the prediction on the input data, the i-th element being the (OOB) error rate for all trees up to the i-th.
- `confusion` — (classification only) the confusion matrix of the prediction (based on OOB data).
- `votes` — (classification only) a matrix with one row for each input data point and one column for each class, giving the fraction or number of (OOB) 'votes' from the random forest.
- `oob.times` — number of times cases are 'out-of-bag' (and thus used in computing OOB error estimate)
- `proximity` — if proximity=TRUE when randomForest is called, a matrix of proximity measures among the input (based on the frequency that pairs of data points are in the same terminal nodes).
- `mse` — (regression only) vector of mean square errors: sum of squared residuals divided by n.
- `rsq` — (regression only) "pseudo R-squared": 1 - mse / Var(y).
- `test` — if test set is given (through the xtest or additionally ytest arguments), this component is a list which contains the corresponding predicted, err.rate, confusion, votes (for classification) or predicted, mse and rsq (for regression) for the test set. If proximity=TRUE, there is also a component, proximity, which contains the proximity among the test set as well as proximity between test and training data.

Note

The forest structure is slightly different between classification and regression. For details on how the trees are stored, see the help page for getTree.

If xtest is given, prediction of the test set is done "in place" as the trees are grown. If ytest is also given, and do.trace is set to some positive integer, then for every do.trace trees, the test set error is printed. Results for the test set is returned in the test component of the resulting randomForest object. For classification, the votes component (for training or test set data) contain the votes the cases received for the classes. If norm.votes=TRUE, the fraction is given, which can be taken as predicted probabilities for the classes.

For large data sets, especially those with large number of variables, calling randomForest via the formula interface is not advised: There may be too much overhead in handling the formula.

The "local" (or casewise) variable importance is computed as follows: For classification, it is the increase in percent of times a case is OOB and misclassified when the variable is permuted. For regression, it is the average increase in squared OOB residuals when the variable is permuted.

Author(s)

Andy Liaw <andy_liaw@merck.com> and Matthew Wiener <matthew_wiener@merck.com>, based on original Fortran code by Leo Breiman and Adele Cutler.

References

Breiman, L. (2001), *Random Forests*, Machine Learning 45(1), 5-32.

Breiman, L (2002), "Manual On Setting Up, Using, And Understanding Random Forests V3.1", https://www.stat.berkeley.edu/~breiman/Using_random_forests_V3.1.pdf.

See Also

predict.randomForest, varImpPlot

Examples

```
## Classification:
##data(iris)
set.seed(71)
iris.rf <- randomForest(Species ~ ., data=iris, importance=TRUE,
                        proximity=TRUE)
print(iris.rf)
## Look at variable importance:
round(importance(iris.rf), 2)
## Do MDS on 1 - proximity:
iris.mds <- cmdscale(1 - iris.rf$proximity, eig=TRUE)
op <- par(pty="s")
pairs(cbind(iris[,1:4], iris.mds$points), cex=0.6, gap=0,
      col=c("red", "green", "blue")[as.numeric(iris$Species)],
      main="Iris Data: Predictors and MDS of Proximity Based on RandomForest")
par(op)
print(iris.mds$GOF)
```

```
## The `unsupervised' case:
set.seed(17)
iris.urf <- randomForest(iris[, -5])
MDSplot(iris.urf, iris$Species)

## stratified sampling: draw 20, 30, and 20 of the species to grow each tree.
(iris.rf2 <- randomForest(iris[1:4], iris$Species,
                          sampsize=c(20, 30, 20)))

## Regression:
## data(airquality)
set.seed(131)
ozone.rf <- randomForest(Ozone ~ ., data=airquality, mtry=3,
                         importance=TRUE, na.action=na.omit)
print(ozone.rf)
## Show "importance" of variables: higher value mean more important:
round(importance(ozone.rf), 2)

## "x" can be a matrix instead of a data frame:
set.seed(17)
x <- matrix(runif(5e2), 100)
y <- gl(2, 50)
(myrf <- randomForest(x, y))
(predict(myrf, x))

## "complicated" formula:
(swiss.rf <- randomForest(sqrt(Fertility) ~ . - Catholic + I(Catholic < 50),
                          data=swiss))
(predict(swiss.rf, swiss))
## Test use of 32-level factor as a predictor:
set.seed(1)
x <- data.frame(x1=gl(53, 10), x2=runif(530), y=rnorm(530))
(rf1 <- randomForest(x[-3], x[[3]], ntree=10))

## Grow no more than 4 nodes per tree:
(treesize(randomForest(Species ~ ., data=iris, maxnodes=4, ntree=30)))

## test proximity in regression
iris.rrf <- randomForest(iris[-1], iris[[1]], ntree=101, proximity=TRUE, oob.prox=FALSE)
str(iris.rrf$proximity)
```

---

rfcv — *Random Forest Cross-Valdidation for feature selection*

Description

This function shows the cross-validated prediction performance of models with sequentially reduced number of predictors (ranked by variable importance) via a nested cross-validation procedure.

Usage

```
rfcv(trainx, trainy, cv.fold=5, scale="log", step=0.5,
     mtry=function(p) max(1, floor(sqrt(p))), recursive=FALSE, ...)
```

Arguments

`trainx`	matrix or data frame containing columns of predictor variables
`trainy`	vector of response, must have length equal to the number of rows in `trainx`
`cv.fold`	number of folds in the cross-validation
`scale`	if `"log"`, reduce a fixed proportion (`step`) of variables at each step, otherwise reduce `step` variables at a time
`step`	if `log=TRUE`, the fraction of variables to remove at each step, else remove this many variables at a time
`mtry`	a function of number of remaining predictor variables to use as the `mtry` parameter in the `randomForest` call
`recursive`	whether variable importance is (re-)assessed at each step of variable reduction
`...`	other arguments passed on to `randomForest`

Value

A list with the following components:

list(n.var=n.var, error.cv=error.cv, predicted=cv.pred)

`n.var`	vector of number of variables used at each step
`error.cv`	corresponding vector of error rates or MSEs at each step
`predicted`	list of `n.var` components, each containing the predicted values from the cross-validation

Author(s)

Andy Liaw

References

Svetnik, V., Liaw, A., Tong, C. and Wang, T., "Application of Breiman's Random Forest to Modeling Structure-Activity Relationships of Pharmaceutical Molecules", MCS 2004, Roli, F. and Windeatt, T. (Eds.) pp. 334-343.

See Also

`randomForest`, `importance`

Examples

```
set.seed(647)
myiris <- cbind(iris[1:4], matrix(runif(96 * nrow(iris)), nrow(iris), 96))
result <- rfcv(myiris, iris$Species, cv.fold=3)
with(result, plot(n.var, error.cv, log="x", type="o", lwd=2))

## The following can take a while to run, so if you really want to try
## it, copy and paste the code into R.

## Not run:
result <- replicate(5, rfcv(myiris, iris$Species), simplify=FALSE)
error.cv <- sapply(result, "[[", "error.cv")
matplot(result[[1]]$n.var, cbind(rowMeans(error.cv), error.cv), type="l",
        lwd=c(2, rep(1, ncol(error.cv))), col=1, lty=1, log="x",
        xlab="Number of variables", ylab="CV Error")

## End(Not run)
```

`rfImpute` *Missing Value Imputations by randomForest*

Description

Impute missing values in predictor data using proximity from randomForest.

Usage

```
## Default S3 method:
rfImpute(x, y, iter=5, ntree=300, ...)
## S3 method for class 'formula'
rfImpute(x, data, ..., subset)
```

Arguments

`x`	A data frame or matrix of predictors, some containing NAs, or a formula.
`y`	Response vector (NA's not allowed).
`data`	A data frame containing the predictors and response.
`iter`	Number of iterations to run the imputation.
`ntree`	Number of trees to grow in each iteration of randomForest.
`...`	Other arguments to be passed to `randomForest`.
`subset`	A logical vector indicating which observations to use.

Details

The algorithm starts by imputing NAs using na.roughfix. Then randomForest is called with the completed data. The proximity matrix from the randomForest is used to update the imputation of the NAs. For continuous predictors, the imputed value is the weighted average of the non-missing obervations, where the weights are the proximities. For categorical predictors, the imputed value is the category with the largest average proximity. This process is iterated iter times.

Note: Imputation has not (yet) been implemented for the unsupervised case. Also, Breiman (2003) notes that the OOB estimate of error from randomForest tend to be optimistic when run on the data matrix with imputed values.

Value

A data frame or matrix containing the completed data matrix, where NAs are imputed using proximity from randomForest. The first column contains the response.

Author(s)

Andy Liaw

References

Leo Breiman (2003). Manual for Setting Up, Using, and Understanding Random Forest V4.0. https://www.stat.berkeley.edu/~breiman/Using_random_forests_v4.0.pdf

See Also

na.roughfix.

Examples

```
data(iris)
iris.na <- iris
set.seed(111)
## artificially drop some data values.
for (i in 1:4) iris.na[sample(150, sample(20)), i] <- NA
set.seed(222)
iris.imputed <- rfImpute(Species ~ ., iris.na)
set.seed(333)
iris.rf <- randomForest(Species ~ ., iris.imputed)
print(iris.rf)
```

rfNews — *Show the NEWS file*

Description

Show the NEWS file of the randomForest package.

Usage

```
rfNews()
```

Value

None.

treesize — *Size of trees in an ensemble*

Description

Size of trees (number of nodes) in and ensemble.

Usage

```
treesize(x, terminal=TRUE)
```

Arguments

`x`	an object of class `randomForest`, which contains a `forest` component.
`terminal`	count terminal nodes only (`TRUE`) or all nodes (`FALSE`

Value

A vector containing number of nodes for the trees in the `randomForest` object.

Note

The `randomForest` object must contain the `forest` component; i.e., created with `randomForest(..., keep.forest=TRU`[illegible]

Author(s)

Andy Liaw <andy_liaw@merck.com>

See Also

randomForest

Examples

```
data(iris)
iris.rf <- randomForest(Species ~ ., iris)
hist(treesize(iris.rf))
```

---

tuneRF *Tune randomForest for the optimal mtry parameter*

Description

Starting with the default value of mtry, search for the optimal value (with respect to Out-of-Bag error estimate) of mtry for randomForest.

Usage

```
tuneRF(x, y, mtryStart, ntreeTry=50, stepFactor=2, improve=0.05,
       trace=TRUE, plot=TRUE, doBest=FALSE, ...)
```

Arguments

x	matrix or data frame of predictor variables
y	response vector (factor for classification, numeric for regression)
mtryStart	starting value of mtry; default is the same as in randomForest
ntreeTry	number of trees used at the tuning step
stepFactor	at each iteration, mtry is inflated (or deflated) by this value
improve	the (relative) improvement in OOB error must be by this much for the search to continue
trace	whether to print the progress of the search
plot	whether to plot the OOB error as function of mtry
doBest	whether to run a forest using the optimal mtry found
...	options to be given to randomForest

Value

If doBest=FALSE (default), it returns a matrix whose first column contains the mtry values searched, and the second column the corresponding OOB error.

If doBest=TRUE, it returns the randomForest object produced with the optimal mtry.

See Also

randomForest

Examples

```
data(fgl, package="MASS")
fgl.res <- tuneRF(fgl[,-10], fgl[,10], stepFactor=1.5)
```

varImpPlot — *Variable Importance Plot*

Description

Dotchart of variable importance as measured by a Random Forest

Usage

```
varImpPlot(x, sort=TRUE, n.var=min(30, nrow(x$importance)),
           type=NULL, class=NULL, scale=TRUE,
           main=deparse(substitute(x)), ...)
```

Arguments

`x`	An object of class `randomForest`.
`sort`	Should the variables be sorted in decreasing order of importance?
`n.var`	How many variables to show? (Ignored if `sort=FALSE`.)
`type, class, scale`	arguments to be passed on to `importance`
`main`	plot title.
`...`	Other graphical parameters to be passed on to `dotchart`.

Value

Invisibly, the importance of the variables that were plotted.

Author(s)

Andy Liaw `<andy\_liaw@merck.com>`

See Also

`randomForest`, `importance`

Examples

```
set.seed(4543)
data(mtcars)
mtcars.rf <- randomForest(mpg ~ ., data=mtcars, ntree=1000, keep.forest=FALSE,
                          importance=TRUE)
varImpPlot(mtcars.rf)
```

varUsed *Variables used in a random forest*

Description

Find out which predictor variables are actually used in the random forest.

Usage

```
varUsed(x, by.tree=FALSE, count=TRUE)
```

Arguments

x	An object of class randomForest.
by.tree	Should the list of variables used be broken down by trees in the forest?
count	Should the frequencies that variables appear in trees be returned?

Value

If count=TRUE and by.tree=FALSE, a integer vector containing frequencies that variables are used in the forest. If by.tree=TRUE, a matrix is returned, breaking down the counts by tree (each column corresponding to one tree and each row to a variable).

If count=FALSE and by.tree=TRUE, a list of integer indices is returned giving the variables used in the trees, else if by.tree=FALSE, a vector of integer indices giving the variables used in the entire forest.

Author(s)

Andy Liaw

See Also

randomForest

Examples

```
data(iris)
set.seed(17)
varUsed(randomForest(Species~., iris, ntree=100))
```

Index

*Topic **NA**
na.roughfix, 10
*Topic **classif**
classCenter, 2
combine, 3
grow, 5
importance, 6
margin, 8
MDSplot, 9
outlier, 11
partialPlot, 12
plot.randomForest, 14
predict.randomForest, 15
randomForest, 17
rfcv, 21
rfImpute, 23
rfNews, 25
treesize, 25
tuneRF, 26
varImpPlot, 27
*Topic **datasets**
imports85, 7
*Topic **regression**
combine, 3
grow, 5
importance, 6
partialPlot, 12
plot.randomForest, 14
predict.randomForest, 15
randomForest, 17
rfcv, 21
rfImpute, 23
treesize, 25
varImpPlot, 27
*Topic **tree**
getTree, 4
importance, 6
MDSplot, 9
partialPlot, 12
plot.randomForest, 14
randomForest, 17
rfImpute, 23
tuneRF, 26
varImpPlot, 27
varUsed, 28

classCenter, 2
cmdscale, *9*
combine, 3, *5*

dotchart, *27*

getTree, 4, *20*
grow, *3*, 5

importance, 6, *22*, *27*
imports85, 7

margin, 8
MDSplot, *3*, 9

na.roughfix, 10, *24*

outlier, 11

pairs, *10*
partialPlot, 12
plot.margin *(*margin*)*, 8
plot.randomForest, 14
predict.randomForest, 15, *20*
print.randomForest *(*randomForest*)*, 17

randomForest, *3–16*, 17, *22–28*
rfcv, 21
rfImpute, *11*, 23
rfNews, 25

treesize, 25
tuneRF, 26

varImpPlot, *7*, *20*, 27
varUsed, 28
